# Older Adults’ Access to Care during the COVID-19 Pandemic: Results from the LOckdown and LifeSTyles (LOST) in Lombardia Project

**DOI:** 10.3390/ijerph191811271

**Published:** 2022-09-07

**Authors:** Giacomo Pietro Vigezzi, Paola Bertuccio, Andrea Amerio, Cristina Bosetti, Davide Gori, Luca Cavalieri d’Oro, Licia Iacoviello, David Stuckler, Alberto Zucchi, Silvano Gallus, Anna Odone, Lost in Lombardia Project Investigators

**Affiliations:** 1 Department of Public Health, Experimental and Forensic Medicine, University of Pavia, 27100 Pavia, Italy; 2 Collegio Ca’ della Paglia, Fondazione Ghislieri, 27100 Pavia, Italy; 3 Department of Neuroscience, Rehabilitation, Ophthalmology, Genetics, Maternal and Child Health (DINOGMI), Section of Psychiatry, University of Genoa, 16124 Genoa, Italy; 4 IRCCS San Martino Polyclinic Hospital, 16132 Genoa, Italy; 5 Department of Oncology, Istituto di Ricerche Farmacologiche Mario Negri IRCCS, 20156 Milan, Italy; 6 Department of Biomedical and Neuromotor Science, University of Bologna, 40126 Bologna, Italy; 7 Epidemiology Unit, Brianza Health Protection Agency, 20900 Monza, Italy; 8 Research Center in Epidemiology and Preventive Medicine (EPIMED), Department of Medicine and Surgery, University of Insubria, 21100 Varese, Italy; 9 Department of Epidemiology and Prevention, IRCCS Neuromed, 86077 Pozzilli, Italy; 10 Department of Social and Political Sciences, Bocconi University, 20100 Milan, Italy; 11 Epidemiology Unit, Bergamo Health Protection Agency, 24121 Bergamo, Italy; 12 Department of Environmental Health Sciences, Istituto di Ricerche Farmacologiche Mario Negri IRCCS, 20156 Milan, Italy

**Keywords:** COVID-19 pandemic, delivery of health care, healthcare equity, access to healthcare, cross-sectional studies, older adults

## Abstract

The COVID-19 pandemic disproportionally affected older people in terms of clinical outcomes and care provision. We aimed to investigate older adults’ changes in access to care during the pandemic and their determinants. We used data from a cross-sectional study (LOST in Lombardia) conducted in autumn 2020 on a representative sample of 4400 older adults from the most populated region in Italy. Lifestyles, mental health, and access to healthcare services before and during the pandemic were collected. To identify factors associated with care delays, reduction in emergency department (ED) access, and hospitalisations, we estimated prevalence ratios (PR) and 95% confidence intervals (CI) using multivariable log-binomial regression models. During the pandemic, compared to the year before, 21.5% of the study population increased telephone contacts with the general practitioner (GP) and 9.6% increased self-pay visits, while 22.4% decreased GP visits, 12.3% decreased outpatient visits, 9.1% decreased diagnostic exams, 7.5% decreased ED access, and 6% decreased hospitalisations. The prevalence of care delays due to patient’s decision (overall 23.8%) was higher among men (PR 1.16, 95% CI 1.05–1.29), subjects aged 75 years or more (PR 1.12, 95% CI 1.00–1.25), and those with a higher economic status (*p* for trend < 0.001). Participants with comorbidities more frequently cancelled visits and reduced ED access or hospitalisations, while individuals with worsened mental health status reported a higher prevalence of care delays and ED access reductions. Access to care decreased in selected sub-groups of older adults during the pandemic with likely negative impacts on mortality and morbidity in the short and long run.

## 1. Introduction

Italy was the first European country hit by the coronavirus disease 2019 (COVID-19) pandemic. The virus started to spread in the Lombardy region, becoming the area with the greatest excess in mortality during the first wave of the pandemic, mainly in people aged 65 or more [1,2]. The emergency overwhelmed the healthcare system, which had to reorganise to face the rapid and steady increase in hospitalisations of infected subjects [3] and the ten-fold growth of emergency requests and events managed [4,5]. The pandemic burden, along with imposed containment measures (e.g., stay-at-home order), affected the older population and jeopardised chronic disease management. This resulted in changes in healthcare service supply and demand [6,7], affecting care provision and outcomes. On the one hand, COVID-19 containment measures caused a reduction in healthcare delivery, whose services were running at stretched capacity [7]; on the other hand, fear of COVID-19 infection discouraged people’s care-seeking for non-COVID-19 medical reasons [8,9].

The use of healthcare services by older people faces several demographic, socioeconomic, and cultural barriers, such as income, education, employment and residence status, digital literacy [10], as well as health status [11]. Mounting evidence has been providing data on changes in healthcare service use during the pandemic [12,13,14]. Still, only a few studies focused on older populations and the determinants of their access to care [15], despite the great need for new evidence to support policymakers and healthcare providers in planning healthcare services for this fragile population [16] and addressing growing inequalities [17].

With the general aim of contributing to fill these gaps in knowledge, we described the impact of the COVID-19 pandemic on older adults’ access to care −from primary care to self-pay consultations− and investigated associated factors, including sociodemographic characteristics, comorbidities, and mental health indicators, using data from a large cross-sectional study conducted in northern Italy [18,19,20,21,22,23].

## 2. Materials and Methods

### 2.1. Study Design

We analysed data from the LOckdown and lifeSTyles (LOST) in Lombardia project, a large cross-sectional study conducted in autumn 2020 on a representative sample of older people in the Italian region of Lombardy. The total study sample included 4400 subjects aged 65 and over, representative by age, gender, and municipality size. This project replicated the nationwide survey named LOST in Italy at the regional level among older adults [24,25].

### 2.2. Data Collection and Measures

The study was conducted in collaboration with Doxa, the Italian branch of the Worldwide Independent Network/Gallup International Association. Participants were selected among the Doxa online panel and were randomly recruited from a list of approximately 30,000 households living in the Lombardy region, representative by province and municipality. A quota method was used to enrol study participants in order to guarantee the sample group’s representativeness, using quotas for gender, age group, and municipality size.

Interviews were carried out on the basis of a structured questionnaire through a computer-assisted telephone interviewing (CATI) method. Participants were asked for information on demographic and socioeconomic characteristics, anthropometric measures (height and weight), lifestyle behaviours (i.e., physical activity, tobacco smoking, alcohol drinking, diet), psychological wellbeing, and their history of chronic diseases. Concerning mental health status, a specific section evaluated sleep quality and quantity, anxiety, and depressive symptoms through validated scales each administered twice with reference to experience both before and during the pandemic. In particular, the presence of anxiety symptoms was assessed using the 2-item Generalised Anxiety Disorder (GAD-2), a short version of the 7-item scale (GAD-7) [26], while depressive symptoms were established through the 2-item Patient Health Questionnaire (PHQ-2), based on the 9-item validated scale (PHQ-9) [27].

To assess the COVID-19 pandemic’s impact on access to care, specific questions were asked with reference to time during the pandemic and the year before (i.e., the baseline). In detail, participants were asked if telephone contact with the general practitioner (GP), visits to a GP, access to the emergency department (ED), hospitalisations, outpatient visits, diagnostic exams, self-pay specialist visits, and medicine purchases with or without medical prescription increased, decreased, or remained unchanged. In addition, care delays (i.e., cancellations or postponements of scheduled visits or surgeries) initiated by the providers or as a consequence of the patient’s decision, and interruptions of treatments for chronic conditions were also assessed with binary questions (yes, no).

### 2.3. Outcomes and Variables of Interest

The primary outcome of interest was care delays due to the patient’s decision during the COVID-19 pandemic. Secondary outcomes were the reduction in ED access and hospitalisations during the COVID-19 pandemic compared to the year before, i.e., autumn 2019.

As potentially associated factors, we considered sociodemographic characteristics, including gender, age group, marital status, educational level, self-reported economic status, number of household components, number of comorbidities, and self-reported COVID-19 infection. For mental health, we considered: anxiety symptoms at baseline (GAD-2 scale value < 3: no, ≥3: yes), change in anxiety symptoms due to the pandemic (unchanged < 3, unchanged ≥3, worsened, improved), depressive symptoms at baseline (PHQ-2 scale value < 3: no, ≥3: yes), and change in depressive symptoms due to the pandemic (unchanged <3, unchanged ≥3, worsened, improved).

### 2.4. Statistical Analysis

Using multivariable log-binomial regression models, we estimated prevalence ratios (PRs) and corresponding 95% confidence intervals (CIs) for the outcomes of interest according to sociodemographic characteristics, comorbidities, and mental health indicators. As a sensitivity analysis, we estimated PRs and 95% CIs among the subgroups of people who reported at least one chronic condition. A statistical weight was applied to all the analyses to generate representative estimates of the elderly population in Lombardy. All statistical analyses were performed using SAS 9.4 (Cary, NC, USA).

## 3. Results

Characteristics of the study population are reported in Table 1. Overall, 57% of the study population were women, 52% were aged 75 and over, and most were married (about 71%), lived with at least another person, and had a secondary or high school level education. Eighty percent of the study population declared at least one chronic disease; among those, about 29% had one chronic disease, 32.5% had two diseases, and about 18% had three or more. Seventeen percent of the participants reported a worsening of anxiety symptoms during the pandemic, whereas 10% reported worsening depressive symptoms.

Table 2 reports the weighted prevalence of chronic diseases and corresponding 95% CIs, reported overall and by gender separately. Hypertension was the most common chronic disease reported in our sample of older adults, with a prevalence of about 56% in both genders. In women, the second most common chronic condition was arthritis (43.2%), followed by osteoporosis (21.7%) and diabetes (18.9%). Among men, diabetes was the second most common disease and more prevalent than in women (24.9%), followed by arthritis (22.7%) and other heart diseases (15.2%).

**Figure 1:**
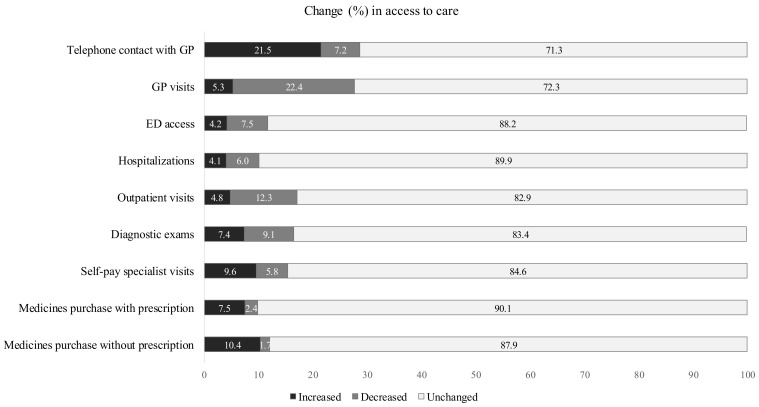
Proportions of change in access to care in the overall study population (*n* = 4400). GP: general practitioner; ED: emergency department.

Figure 1 shows the proportions of subjects who increased, decreased, or did not change their demand for care during the pandemic, compared to the year before. More than 20% increased telephone contacts with their GP and decreased GP visits. About 7.5% reduced access to ED, 6% reduced hospitalisations, 12.3% reduced outpatient visits, 9.1% reduced diagnostic exams, while 9.6% increased self-pay specialist visits, 7.5% increased medicine purchases with a prescription, and 10.4% without a prescription. In addition, 35% of participants reported care delays due to providers’ decisions, while 23.8% were due to their own decision; 9.4% reported disruptions in ongoing treatments for chronic conditions.

Table 3 reports the prevalence and adjusted PRs for the outcomes of interest according to sociodemographic characteristics, the number of comorbidities, and mental health indicators. The overall prevalence of care delays due to the patient’s decision was 23.8%. This prevalence was higher among men than women (PR 1.16, 95% CI 1.05–1.29) and among subjects aged 75 or over than those aged 65–74 (PR 1.12, 95% CI 1.00–1.25). There was a positive association between care delays and the self-reported economic status (PR 1.83, 95% CI 1.49–2.25 for high compared to low status) with a significant linear trend (*p* < 0.001), and between care delays and number of comorbidities (PR 2.74, 95% CI 2.23–3.36 for people with three or more chronic diseases compared to healthy ones, *p* for trend <0.001). Individuals who reported worse anxiety and depressive symptoms had a higher prevalence of care delays than those without symptoms before and after the pandemic, with a PR of 1.19 (95% CI 1.05–1.36) for anxiety and 1.21 (95% CI 1.04–1.41) for depression. The reduction in ED access and hospitalisations was positively associated with older age, number of household members, and number of comorbidities. In contrast, the ED access reduction was inversely associated with self-reported economic status.

The results remained consistent, when restricting the analyses to the subgroup of 3521 subjects who reported at least one chronic disease (Table S1).

## 4. Discussion

This study provides an informative perspective on the patterns of change in access to care during the first phases of the COVID-19 pandemic and associated factors, in a large representative sample of older individuals in northern Italy. On the one hand, GP visits, ED access, hospitalisations, outpatient visits, and the request for diagnostic exams decreased to more than 22% of older adults during the COVID-19 emergency compared to pre-pandemic times. On the other hand, telephone contacts with a GP, self-pay specialist visits, as well as medicine purchases with or without a prescription increased to more than 20%. In addition, about 24% of older adults reported care delays during the COVID-19 pandemic; this value would be 27% if we consider only people with at least one chronic disease.

With reference to determinants of decreased access to care during the COVID-19 crisis, men had a higher prevalence of care delays compared to women; the oldest adults reported a higher prevalence of care delays and reductions in ED access and hospitalisations. A higher number of household members was associated with a lower prevalence of care delays and a higher prevalence of decreased ED access and hospitalisations. A higher self-reported economic status was associated with an increased prevalence of care delays and a decreased prevalence of reduction in ED access, with a significant trend. Regarding comorbidities, we report an increasing trend in the prevalence of care delays, but also in the reduction in ED access and hospitalisations as the number of chronic diseases grows. In addition, older adults who reported worsening anxiety and depressive symptoms during the pandemic had a higher prevalence of care delays and reduction in ED access than those who did not develop symptoms before and during the pandemic.

The COVID-19 pandemic disproportionally impacted older adults and even more so, those affected by chronic diseases. Not only were they the most at-risk for severe COVID-19 clinical forms, complications and death, showing the highest mortality rates worldwide [28], but they also suffered from emergency restrictions and healthcare system downscaling, closures, and remodelling with unmet demands for healthcare services other than COVID-19 [2]. During the first COVID-19 epidemic wave, policymakers’ decisions on which services to keep in operation and the duration of restrictions varied widely among countries [29]. A generalised reduction in regular healthcare provision was put in place for hospital-centred, outpatient, and primary care levels. As confirmed by our results, both ED access and hospital admissions decreased significantly by about 30% during the pandemic [5,30], despite the massive efforts deployed to admit COVID-19 patients needing intensive care [31,32]. Similarly, considerable reductions in cancer referrals [33] and diagnoses [34] were extensively reported. Healthcare providers reorganised services and waiting lists in response: routine follow-ups were cancelled and first-time outpatient consultations were prioritised [35].

In line with the available evidence, we observed the most significant impact on primary care [36,37]. It emerged that GPs had to change their activities to face the pandemic and guarantee the most suitable offer to their patients. Nonetheless, just as it occurred in our sample, part of the Italian population turned to private healthcare providers for services that the public sector could not provide [2,38].

Since the observed downscaling of the healthcare system contributed to the exacerbation of inequalities [17], identifying the determinants that pose a higher risk for healthcare access discontinuity is crucial to inform future health policy planning, implementation, and evaluation. Several studies provided data on the changes in access to care during the global COVID-19 health emergency [7,39], but only a few investigated associated factors [8,9,15,40,41,42] and none were conducted among older adults. A study combining data from 25 European countries and Israel on people aged 50 or more found that women, more educated and occupationally active subjects and those living in urban areas were more likely to experience limited access to healthcare [43]. Our findings can be interpreted in light of both individual- and provider-level characteristics, such as gender, age, socioeconomic status (SES), and mental health status [44], but also health policies and system structure influencing older adults’ access to care during COVID-19 [15].

Concerning demographic characteristics, conversely to our results, in other European countries a higher prevalence of healthcare avoidance was found among women compared to men [8], and this was also found in other areas of the world [40,45]. Yet, the main reasons hypothesised for the reduction in access to care (i.e., job insecurity, overlapping responsibilities in work, domestic labour, and family dimensions) are lacking among older female subjects in Italy, who are among the least employed group in Europe as they are entirely committed to their families. Therefore, the observed gender inequalities in access to care may reflect differences in health-related behaviours, health and risk perception, as demonstrated in studies conducted both before and during the COVID-19 pandemic [9,46]: women are more likely than men to engage in health behaviours and care adherence [47]. As for age, our results suggest that older individuals might have avoided access to care because of the fear of nosocomial SARS-CoV-2 transmission given their higher risk of death, even if they were in need [48].

The role of SES, including education, as a determinant of access to care has already emerged in several studies on the general population before the pandemic [49], and our results confirmed this association. We found significant trends in the association between self-reported SES and care delays, as well as reductions in ED access. A possible explanation is that high-SES older people, perhaps being more aware of the healthcare demands arising from COVID-19 infected patients, tended to limit their routine care [43], having the possibility to turn up to private healthcare providers and thus, avoid waiting lists [38,45]. Other studies reported that people with higher SES were more likely to renounce healthcare due to fear of infection, even though this population claimed fewer health concerns than lower educated individuals during the pandemic [36,50].

Larger household size was associated with a higher prevalence of a reduction in ED access and hospital admissions in our sample. Those living alone might have searched for more help from healthcare facilities, lacking the support of other relatives or caregivers during national lockdown [25]. Loneliness is a paramount public health determinant, especially among older people, and a risk factor for health, linked to increased healthcare utilisation [51].

Avoiding healthcare centres could be considered a COVID-19 preventive behaviour, and participants reporting poorer health were more likely to refuse to seek care, even when in need [52]. Two hypotheses can be considered to explain the higher prevalence of avoidance behaviours. First, people with pre-existing comorbidities felt more at risk of severe COVID-19 outcomes [53]; second, chronic patients generally have more scheduled consultations, and these were cancelled when healthcare facilities reallocated resources for COVID-19 patients or were voluntarily postponed [36]. Decreases in physician consultations, specialist referrals, and hospital admissions observed among older people managing comorbidities in different countries [54,55] caused a decline in the diagnosis of incident diseases, with remarkable potential consequences for health in the medium and long term.

Consistent evidence exists on psychological determinants as drivers associated with changes in access to care. In our sample, a worsening in anxiety or depressive symptoms was associated with a higher prevalence of care delays during the pandemic. Similarly, a cohort study conducted in Hong Kong found that mental health problems were more frequent in older people with comorbidities who missed medical appointments for chronic disease care [42]. Available evidence endorses the prevalence of avoidance of healthcare assistance to be higher in people who experienced more anxiety and depressive symptoms [8,9,41].

Our study needs to be interpreted in light of several strengths and limitations. To our knowledge, the LOST in Lombardia project is the first multidisciplinary study conducted on a large representative sample exploring the effects of the pandemic on various health and healthcare-related outcomes in a region of 10 million inhabitants at the heart of the COVID-19 outbreak in Europe. These characteristics (i.e., large sample size and its representativeness of the general elderly population) allow us to generalise our results to other countries with publicly funded healthcare systems. In addition, this study enabled us to obtain the estimates of the prevalence of the most common chronic diseases, which are the latest available for the Lombardy region, not included in the PASSI d’Argento surveillance [56]. Given that, except for diabetes, all the chronic diseases were less prevalent in Lombardy than in Italy among people over 65, our estimates highlight health inequalities across Italian regions [57]. The differences among regional healthcare systems support the role of wealth and service availability in maintaining collective health [58,59], especially in an ageing population requiring routine medical assistance with a massive increase in associated demand and costs [60].

The adopted study design acknowledged simulating a pre-post analysis in the context of a cross-sectional study, exploiting the COVID-19 emergency as a quasi-natural experiment [61]. Potential selection bias was overcome using the CATI method, which is the most suitable survey method for our sample of older subjects who generally have little confidence with digital devices. Finally, validated evidence-based tools ensured a rigorous assessment of the collected variables.

Concerning limitations, the cross-sectional nature of our data does not allow us to infer robust causality for the observed outcomes. The longitudinal follow-up might therefore consolidate our results. Other limitations include the possible information bias due to self-reported responses and a potential social desirability recall bias since participants were asked to report their habits and psychophysical indicators before the pandemic at the time of the interview. Nursing home and long-term care residents were not included in the population sample.

Overall, gaps in our knowledge persist. More extended longitudinal studies might help measure the impact of decreasing medical follow-up for people with chronic diseases. As public health representatives, we claim to investigate the long-term consequences on the trust in healthcare services and workers: the imposed closures could have been harmful, particularly for routine childhood immunisation services [62,63] and screening programmes.

Further exploration of the underlying reasons and determinants of avoiding or delaying access to care among the most vulnerable groups is mandatory for epidemic preparedness. Since disadvantaged people with poorer health appear to be the most at-risk, healthcare discontinuities might deepen existing inequalities, which, among the older population, include the risk of exclusion from telehealth services [64]. During the pandemic, a wide range of digital-based services was leveraged, for instance, for patients unable to attend in-person appointments. Still, digital literacy gaps could be an obstacle to comprehensive access to services, which help prevent the delay of much-needed care [10].

## 5. Conclusions

The decrease in healthcare provision and medical consultations observed during the pandemic could result in indirect adverse health outcomes, including complications of chronic conditions, hospitalisations, and deaths, especially for old, fragile adults [65,66]. Most of the reported care delays were determined by healthcare providers cancelling appointments [36], highlighting health system challenges in supplying routine care during health emergencies. Multisectorial efforts are needed to maintain essential care and optimise scarce resources when demand rises, improving the system’s flexibility, as well as health infrastructures’ accessibility during the pandemic. Public health and primary care services, often inadequately funded, could play a crucial role in guaranteeing the dual delivery of services to COVID-19 and other patients across levels of care (primary, secondary, tertiary) and settings (inpatient versus long-term care) [67]. Their assets are challenging to expand and strengthen: on-the-field awareness of local context and the reinforcing care delivery of public health and primary care must be pursued long-term [29].

## Figures and Tables

**Table 1:** Distribution of the overall study population (*n* = 4400) according to sociodemographic characteristics, number of chronic diseases, self-reported COVID-19 infection, and mental health indicators.

	Unweighted		Weighted	
	*n*	%	*n*	%
**Gender**				
Women	2537	57.7	2498.0	56.8
Men	1863	42.3	1902.0	43.2
**Age group**				
65–74	2051	46.6	2127.0	48.3
≥75	2349	53.4	2273.0	51.7
**Marital status**				
Married	3092	70.3	3113.1	70.8
Divorced/separated	138	3.1	139.9	3.2
Widowed	896	20.4	870.9	19.8
Single	274	6.2	276.1	6.3
**No. of household components**				
1 (living alone)	996	22.6	975.3	22.2
2	2636	59.9	2646.1	60.1
≥3	768	17.5	778.6	17.7
**Education level**				
None or primary school	809	18.4	788.0	17.9
Secondary	1524	34.6	1521.9	34.6
High school	1627	37.0	1645.7	37.4
University degree	440	10.0	444.5	10.1
**Self-reported economic status**				
Above the average	388	8.8	391.3	8.9
On average	3299	75.0	3303.7	75.1
Below the average	713	16.2	705.0	16.0
**Number of chronic comorbidities**				
0	879	20.0	890.2	20.2
1	1285	29.2	1292.4	29.4
2	1436	32.6	1427.7	32.5
≥3	800	18.2	789.8	17.9
**COVID-19 infection**				
Yes	212	4.8	213.2	4.9
No	4188	95.2	4186.8	95.1
**Anxiety symptoms (GAD-2 scale) in 2019**				
No (<3)	3890	88.4	3892.6	88.5
Yes (≥3)	510	11.6	507.4	11.5
**Change in anxiety symptoms (GAD-2 scale) 2020 vs. 2019**				
Unchanged (<3 in both periods)	3142	71.4	3146.1	71.5
Unchanged (≥3 in both periods)	449	10.2	446.4	10.1
Worsened	748	17.0	746.5	17.0
Improved	61	1.4	61.0	1.4
**Depressive symptoms (PHQ-2 scale) in 2019**				
No (<3)	4054	92.1	4055.6	92.2
Yes (≥3)	346	7.9	344.4	7.8
**Change in depressive symptoms (PHQ-2 scale) 2020 vs. 2019**				
Unchanged (<3 in both periods)	3614	82.1	3616.2	82.2
Unchanged (≥3 in both periods)	289	6.6	287.0	6.5
Worsened	440	10.0	439.4	10.0
Improved	57	1.3	57.4	1.3

GAD-2: Generalised Anxiety Disorder; PHQ-2: Patient Health Questionnaire.

**Table 2:** Weighted prevalence and corresponding 95% confidence intervals (CI) of chronic diseases in the study population, reported overall and by gender.

	Overall *n* = 4400	Men *n* = 1863	Women *n* = 2537
	Prevalence (95% CI)	Prevalence (95% CI)	Prevalence (95% CI)
Hypertension	56.0 (53.5–58.5)	56.7 (54.2–59.2)	55.5 (53.3–57.7)
Osteoarthritis, arthritis	34.3 (32.8–35.8)	22.7 (21.7–23.7)	43.2 (41.5–44.9)
Diabetes	21.5 (20.6–22.4)	24.9 (23.8–26.0)	18.9 (18.2–19.6)
Osteoporosis	15.2 (14.5–15.9)	6.7 (6.4–7.0)	21.7 (20.9–22.5)
Other heart diseases	12.7 (12.2–13.2)	15.2 (14.5–15.9)	10.8 (10.4–11.2)
Chronic bronchitis	4.5 (4.3–4.7)	5.0 (4.8–5.2)	4.1 (4.0–4.2)
Cancer	4.0 (3.8–4.2)	3.8 (3.7–3.9)	4.2 (4.1–4.3)
Asthma	3.4 (3.3–3.5)	3.1 (3.0–3.2)	3.7 (3.6–3.8)
Kidney failure	2.2 (2.1–2.3)	2.3 (2.2–2.4)	2.2 (2.1–2.3)

**Table 3:** Weighted prevalence (Prev.) and unweighted number (*n*) of the change in three selected healthcare service demands according to selected determinants, adjusted prevalence ratios (PR) and corresponding 95% confidence intervals (CI).

	Cancellation/Postponement of Scheduled Visits		Reduction in ED Access		Reduction in Hospitalisations	
	Prev. (*n*)	PR ^1^ (95% CI)	Prev. (*n*)	PR ^1^ (95% CI)	Prev. (*n*)	PR ^1^ (95% CI)
**Overall**	23.8 (1047)	-	7.5 (332)	-	6.0 (263)	-
**Gender**						
Women	22.6 (578)	1^2^	7.2 (186)	1^2^	5.6 (145)	1^2^
Men	25.2 (469)	**1.16 (1.05–1.29)**	7.9 (146)	1.15 (0.93–1.42)	6.4 (118)	1.17 (0.92–1.49)
**Age group**						
65–74	21.4 (439)	1^2^	5.9 (121)	1^2^	4.6 (95)	1^2^
≥75	26.0 (608)	**1.12 (1.00–1.25)**	9.0 (211)	**1.60 (1.27–2.01)**	7.2 (168)	**1.69 (1.30–2.18)**
**Marital status**						
Divorced/widowed/single	23.9 (314)	1^2^	7.0 (93)	1^2^	5.5 (73)	1^2^
Married	23.7 (733)	1.09 (0.90–1.33)	7.7 (239)	0.88 (0.63–1.23)	6.1 (190)	0.84 (0.57–1.23)
**Number of household components**						
1 (living alone)	25.3 (253)	1^2^	6.0 (61)	1^2^	4.6 (47)	1^2^
2	24.5 (647)	0.90 (0.73–1.12)	7.3 (194)	**1.57 (1.05–2.35)**	5.9 (156)	**1.67 (1.06–2.65)**
≥3	19.1 (147)	0.78 (0.61–1.00)	10.0 (77)	**2.44 (1.59–3.74)**	7.8 (60)	**2.49 (1.53–4.05)**
*p for trend*		***0.031***		***<0.001***		***<0.001***
**Education level**						
None or primary school	23.8 (193)	1^2^	9.1 (74)	1^2^	6.5 (53)	1^2^
Secondary	22.9 (349)	1.01 (0.86–1.18)	6.8 (104)	0.79 (0.59–1.06)	5.7 (87)	0.93 (0.66–1.30)
High school/degree	24.3 (505)	1.09 (0.94–1.27)	7.4 (154)	0.94 (0.71–1.26)	5.9 (123)	0.97 (0.69–1.35)
*p for trend*		*0.181*		*0.652*		*0.936*
**Self-reported economic status**						
Low	20.6 (148)	1^2^	10.6 (76)	1^2^	6.5 (47)	1^2^
Medium	23.4 (773)	**1.29 (1.10–1.51)**	6.9 (228)	**0.67 (0.52–0.87)**	5.7 (188)	0.90 (0.66–1.25)
High	32.4 (126)	**1.83 (1.49–2.25)**	7.2 (28)	**0.69 (0.45–1.06)**	7.3 (28)	1.14 (0.71–1.82)
*p for trend*		***<0.001***		***0.016***		*0.775*
**Number of comorbidities**						
0	12.1 (107)	1^2^	5.7 (50)	1^2^	5.3 (46)	1^2^
1	20.9 (267)	**1.75 (1.42–2.15)**	7.0 (90)	1.21 (0.87–1.69)	5.4 (69)	1.01 (0.70–1.45)
2	28.9 (414)	**2.52 (2.07–3.07)**	7.6 (109)	1.33 (0.96–1.84)	5.5 (79)	1.02 (0.71–1.46)
≥3	32.3 (259)	**2.74 (2.23–3.36)**	10.2 (83)	**1.76 (1.24–2.49)**	8.5 (69)	**1.57 (1.09–2.28)**
*p for trend*		***<0.001***		***0.001***		***0.020***
**COVID-19 infection**						
No	23.8 (997)	1^2^	7.4 (312)	1^2^	5.9 (247)	1^2^
Yes	23.7 (50)	1.07 (0.84–1.36)	9.4 (20)	1.30 (0.85–2.00)	7.6 (16)	1.33 (0.82–2.14)
**Anxiety symptoms (GAD-2) in 2019**						
No (<3)	23.6 (919)	1^2^	7.5 (292)	1^2^	5.9 (230)	1^2^
Yes (≥3)	25.1 (128)	1.02 (0.86–1.20)	7.8 (40)	1.00 (0.73–1.38)	6.4 (33)	1.05 (0.73–1.51)
**Change in anxiety symptoms 2020 vs. 2019**						
Unchanged (<3 in both periods)	22.7 (713)	1^2^	7 (220)	1^2^	5.6 (176)	1^2^
Unchanged (≥3 in both periods)	25.6 (115)	1.07 (0.90–1.27)	6.9 (31)	0.91 (0.63–1.32)	6 (27)	1.03 (0.68–1.54)
Worsened	27.2 (206)	**1.19 (1.05–1.36)**	9.5 (72)	**1.31 (1.01–1.69)**	7.1 (54)	1.25 (0.92–1.68)
Improved	21.5 (13)	1.01 (0.62–1.63)	14.8 (9)	**1.96 (1.06–3.60)**	9.8 (6)	1.70 (0.78–3.71)
**Depressive symptoms (PHQ-2) in 2019**						
No (<3)	23.7 (964)	1^2^	7.6 (311)	1^2^	6.1 (247)	1^2^
Yes (≥3)	24.0 (83)	0.96(0.78–1.18)	6.0 (21)	0.76 (0.49–1.18)	4.6 (16)	0.70 (0.42–1.18)
**Change in depressive symptoms 2020 vs. 2019**						
Unchanged (<3 in both periods)	23.0 (833)	1^2^	7.5 (271)	1^2^	6 (217)	1^2^
Unchanged (≥3 in both periods)	24.2 (70)	0.98 (0.79–1.22)	5.4 (16)	0.70 (0.43–1.16)	4.1 (12)	0.63 (0.35–1.14)
Worsened	29.6 (131)	**1.21 (1.04–1.41)**	9.0 (40)	1.18 (0.86–1.62)	6.7 (30)	1.12 (0.77–1.63)
Improved	22.8 (13)	1.02 (0.63–1.67)	8.5 (5)	1.17 (0.49–2.75)	7.1 (4)	1.15 (0.44–2.98)

GAD-2: Generalised Anxiety Disorder; PHQ-2: Patient Health Questionnaire. ^1^ Estimated through weighted log-binomial regression models adjusted by gender, age group, marital status, education level, number of household components, self-reported economic status, number of chronic comorbidities, and self-reported COVID-19 infection. Estimates in bold are statistically significant at 0.05 level. ^2^ Reference category.

## Data Availability

The datasets supporting the conclusions of this study are available from the corresponding author upon request.

## Supplementary Materials

The following supporting information can be downloaded at: https://www.mdpi.com/article/10.3390/ijerph191811271/s1, Table S1: Prevalence of healthcare demand behaviour according to selected characteristics, adjusted prevalence ratios (PRs) and corresponding 95% confidence intervals (CI)*, among the subgroup of 3521 subjects with at least one chronic condition.

## Abbreviations

CATI	computer-assisted telephone interviewing
CIs	confidence intervals
ED	emergency department
GAD	generalised anxiety disorder
GP	general practitioner
LOST	LOckdown and lifeSTyles
PHQ	patient health questionnaire
PRs	prevalence ratios
SES	socioeconomic status

## Appendix A

A Amerio (University of Genoa, Department of Neuroscience, Rehabilitation, Ophthalmology, Genetics, Maternal and Child Health, Section of Psychiatry, Genoa, Italy; IRCCS San Martino Polyclinic Hospital, Genoa, Italy), M Amore (University of Genoa, Department of Neuroscience, Rehabilitation, Ophthalmology, Genetics, Maternal and Child Health, Section of Psychiatry, Genoa, Italy), P Bertuccio (University of Pavia, Department of Public Health, Experimental and Forensic Medicine, Pavia, Italy), M Bonaccio (University of Insubria, Department of Medicine and Surgery, Research Center in Epidemiology and Preventive Medicine, Varese, Italy; IRCCS Neuromed, Department of Epidemiology and Prevention, Pozzilli, Italy), C Bosetti (Istituto di Ricerche Farmacologiche Mario Negri IRCCS, Department of Oncology, Milan, Italy), L Cavalieri d’Oro (Brianza Health Protection Agency, Epidemiology Unit, Monza, Italy), R Ciampichini (Bergamo Health Protection Agency, Epidemiology Unit, Bergamo, Italy), R De Sena (Bocconi University, Department of Social and Political Sciences, Milan, Italy), S Gallus (Istituto di Ricerche Farmacologiche Mario Negri IRCCS, Department of Environmental Health Sciences, Milan, Italy), F Gianfagna (University of Insubria, Department of Medicine and Surgery, Research Center in Epidemiology and Preventive Medicine, Varese, Italy), S Ghislandi (Bocconi University, Department of Social and Political Sciences, Milan, Italy), A Ghulam (University of Insubria, Department of Medicine and Surgery, Research Center in Epidemiology and Preventive Medicine, Varese, Italy), L Iacoviello (University of Insubria, Department of Medicine and Surgery, Research Center in Epidemiology and Preventive Medicine, Varese, Italy; IRCCS Neuromed, Department of Epidemiology and Prevention, Pozzilli, Italy), CM Jarach (Istituto di Ricerche Farmacologiche Mario Negri IRCCS, Department of Environmental Health Sciences, Milan, Italy), A Lugo (Istituto di Ricerche Farmacologiche Mario Negri IRCCS, Department of Environmental Health Sciences, Milan, Italy), G Mosconi (University of Pavia, Department of Public Health, Experimental and Forensic Medicine, Pavia, Italy), A Odone (University of Pavia, Department of Public Health, Experimental and Forensic Medicine, Pavia, Italy), M Rognoni (Brianza Health Protection Agency, Epidemiology Unit, Monza, Italy), G Serafini (University of Genoa, Department of Neuroscience, Rehabilitation, Ophthalmology, Genetics, Maternal and Child Health, Section of Psychiatry, Genoa, Italy), M Scala (Brianza Health Protection Agency, Epidemiology Unit, Monza, Italy), C Signorelli (Vita-Salute San Raffaele University, School of Medicine, Milan, Italy), C Stival (Istituto di Ricerche Farmacologiche Mario Negri IRCCS, Department of Environmental Health Sciences, Milan, Italy), D Stuckler (Bocconi University, Department of Social and Political Sciences, Milan, Italy), GP Vigezzi (University of Pavia, Department of Public Health, Experimental and Forensic Medicine, Pavia, Italy; Collegio Ca’ della Paglia, Fondazione Ghislieri, Pavia, Italy), Y Wang (Bocconi University, Department of Social and Political Sciences, Milan, Italy), A Zucchi (Bergamo Health Protection Agency, Epidemiology Unit, Bergamo, Italy).

## References

[1] Grande E., Fedeli U., Pappagallo M., Crialesi R., Marchetti S., Minelli G., Iavarone I., Frova L., Onder G., Grippo F. (2022). Variation in Cause-Specific Mortality Rates in Italy during the First Wave of the COVID-19 Pandemic: A Study Based on Nationwide Data. Int. J. Environ. Res. Public Health.

[2] Odone A., Delmonte D., Gaetti G., Signorelli C. (2021). Doubled mortality rate during the COVID-19 pandemic in Italy: Quantifying what is not captured by surveillance. Public Health.

[3] Grasselli G., Pesenti A., Cecconi M. (2020). Critical Care Utilization for the COVID-19 Outbreak in Lombardy, Italy: Early Experience and Forecast During an Emergency Response. JAMA.

[4] Fagoni N., Perone G., Villa G.F., Celi S., Bera P., Sechi G.M., Mare C., Zoli A., Botteri M. (2021). The Lombardy Emergency Medical System Faced with COVID-19: The Impact of Out-of-Hospital Outbreak. Prehosp. Emerg. Care.

[5] Stirparo G., Oradini-Alacreu A., Migliori M., Villa G.F., Botteri M., Fagoni N., Signorelli C., Sechi G.M., Zoli A. (2022). Public health impact of the COVID-19 pandemic on the emergency healthcare system. J. Public Health.

[6] Corrao G., Cantarutti A., Monzio Compagnoni M., Franchi M., Rea F. (2021). Change in healthcare during COVID-19 pandemic was assessed through observational designs. J. Clin. Epidemiol..

[7] Moynihan R., Sanders S., Michaleff Z.A., Scott A.M., Clark J., To E.J., Jones M., Kitchener E., Fox M., Johansson M. (2021). Impact of COVID-19 pandemic on utilisation of healthcare services: A systematic review. BMJ Open.

[8] Soares P., Leite A., Esteves S., Gama A., Laires P.A., Moniz M., Pedro A.R., Santos C.M., Goes A.R., Nunes C. (2021). Factors Associated with the Patient’s Decision to Avoid Healthcare during the COVID-19 Pandemic. Int. J. Environ. Res. Public Health.

[9] Lu P., Kong D., Shelley M. (2021). Risk Perception, Preventive Behavior, and Medical Care Avoidance among American Older Adults During the COVID-19 Pandemic. J. Aging Health.

[10] Odone A., Gianfredi V., Sorbello S., Capraro M., Frascella B., Vigezzi G.P., Signorelli C. (2021). The Use of Digital Technologies to Support Vaccination Programmes in Europe: State of the Art and Best Practices from Experts’ Interviews. Vaccines.

[11] Allin S., Grignon M., Le Grand J. (2010). Subjective unmet need and utilization of health care services in Canada: What are the equity implications?. Soc. Sci. Med..

[12] Podubinski T., Townsin L., Thompson S.C., Tynan A., Argus G. (2021). Experience of Healthcare Access in Australia during the First Year of the COVID-19 Pandemic. Int. J. Environ. Res. Public Health.

[13] Odone A., Galea S., Stuckler D., Signorelli C. (2020). The first 10,000 COVID-19 papers in perspective: Are we publishing what we should be publishing?. Eur. J. Public Health.

[14] Makiyama K., Kawashima T., Nomura S., Eguchi A., Yoneoka D., Tanoue Y., Kawamura Y., Sakamoto H., Gilmour S., Shi S. (2021). Trends in Healthcare Access in Japan during the First Wave of the COVID-19 Pandemic, up to June 2020. Int. J. Environ. Res. Public Health.

[15] Bastani P., Mohammadpour M., Samadbeik M., Bastani M., Rossi-Fedele G., Balasubramanian M. (2021). Factors influencing access and utilization of health services among older people during the COVID-19 pandemic: A scoping review. Arch. Public Health.

[16] Pujolar G., Oliver-Anglès A., Vargas I., Vázquez M.-L. (2022). Changes in Access to Health Services during the COVID-19 Pandemic: A Scoping Review. Int. J. Environ. Res. Public Health.

[17] Horton R. (2020). Offline: COVID-19 is not a pandemic. Lancet.

[18] Wang Y., Lugo A., Amerio A., d’Oro L.C., Iacoviello L., Odone A., Zucchi A., Gallus S., Stuckler D. (2022). The Impact of COVID-19 Lockdown Announcements on Mental Health: Quasi-Natural Experiment in Lombardy, Italy. Eur. J. Public Health.

[19] Stival C., Lugo A., Bosetti C., Amerio A., Serafini G., Cavalieri d’Oro L., Odone A., Stuckler D., Iacoviello L., Bonaccio M. (2022). COVID-19 confinement impact on weight gain and physical activity in the older adult population: Data from the LOST in Lombardia study. Clin. Nutr. ESPEN.

[20] Jarach C.M., Lugo A., Stival C., Bosetti C., Amerio A., Cavalieri d’Oro L., Iacoviello L., Odone A., Stuckler D., Zucchi A. (2022). The Impact of COVID-19 Confinement on Tinnitus and Hearing Loss in Older Adults: Data From the LOST in Lombardia Study. Front. Neurol..

[21] Bonaccio M., Gianfagna F., Stival C., Amerio A., Bosetti C., Cavalieri d’Oro L., Odone A., Stuckler D., Zucchi A., Gallus S. (2022). Changes in a Mediterranean lifestyle during the COVID-19 pandemic among elderly Italians: An analysis of gender and socioeconomic inequalities in the “LOST in Lombardia” study. Int. J. Food Sci. Nutr..

[22] Vigezzi G.P., Bertuccio P., Bossi C.B., Amerio A., d’Oro L.C., Derosa G., Iacoviello L., Stuckler D., Zucchi A., Lugo A. (2022). COVID-19 pandemic impact on people with diabetes: Results from a large representative sample of Italian older adults. Prim Care Diabetes.

[23] Bosetti C., Rognoni M., Ciampichini R., Paroni L., Scala M., d’Oro L.C., Zucchi A., Amerio A., Iacoviello L., Ghislandi S. (2022). A real world analysis of COVID-19 impact on hospitalizations in older adults with chronic conditions from an Italian region. Sci. Rep..

[24] Amerio A., Lugo A., Stival C., Fanucchi T., Gorini G., Pacifici R., Odone A., Serafini G., Gallus S. (2021). COVID-19 lockdown impact on mental health in a large representative sample of Italian adults. J. Affect. Disord..

[25] Zeduri M., Vigezzi G.P., Carioli G., Lugo A., Stival C., Amerio A., Gorini G., Pacifici R., Politi P., Gallus S. (2022). COVID-19 lockdown impact on familial relationships and mental health in a large representative sample of Italian adults. Soc. Psychiatry Psychiatr. Epidemiol..

[26] Kroenke K., Spitzer R.L., Williams J.B., Monahan P.O., Löwe B. (2007). Anxiety disorders in primary care: Prevalence, impairment, comorbidity, and detection. Ann. Intern. Med..

[27] Kroenke K., Spitzer R.L., Williams J.B. (2003). The Patient Health Questionnaire-2: Validity of a two-item depression screener. Med. Care.

[28] Islam N., Shkolnikov V.M., Acosta R.J., Klimkin I., Kawachi I., Irizarry R.A., Alicandro G., Khunti K., Yates T., Jdanov D.A. (2021). Excess deaths associated with covid-19 pandemic in 2020: Age and sex disaggregated time series analysis in 29 high income countries. BMJ.

[29] Sagan A., Webb E., Azzopardi-Muscat N., de la Mata I., McKee M., Figueras J., European Observatory on Health Systems and Policies (2021). Health Systems Resilience during COVID-19: Lessons for Building Back Better.

[30] Basis F., Zeidani H., Hussein K., Hareli S. (2022). Drastic Reduction Inpatient Visits to the Emergency Department in a Hospital in Israel During the COVID-19 Outbreak, Compared to the H1N1 2009. Int. J. Health Policy Manag..

[31] Pecoraro F., Luzi D., Clemente F. (2021). Spatial Inequity in Access to Intensive Care Unit Beds at Regional Level in Italy. Stud. Health Technol. Inf..

[32] Zangrillo A., Beretta L., Silvani P., Colombo S., Scandroglio A.M., Dell’Acqua A., Fominskiy E., Landoni G., Monti G., Azzolini M.L. (2020). Fast reshaping of intensive care unit facilities in a large metropolitan hospital in Milan, Italy: Facing the COVID-19 pandemic emergency. Crit. Care Resusc..

[33] Morris E.J.A., Goldacre R., Spata E., Mafham M., Finan P.J., Shelton J., Richards M., Spencer K., Emberson J., Hollings S. (2021). Impact of the COVID-19 pandemic on the detection and management of colorectal cancer in England: A population-based study. Lancet Gastroenterol. Hepatol..

[34] Dinmohamed A.G., Visser O., Verhoeven R.H.A., Louwman M.W.J., van Nederveen F.H., Willems S.M., Merkx M.A.W., Lemmens V., Nagtegaal I.D., Siesling S. (2020). Fewer cancer diagnoses during the COVID-19 epidemic in the Netherlands. Lancet Oncol..

[35] Jereczek-Fossa B.A., Pepa M., Marvaso G., Bruni A., Buglione di Monale E.B.M., Catalano G., Filippi A.R., Franco P., Gambacorta M.A., Genovesi D. (2020). COVID-19 outbreak and cancer radiotherapy disruption in Italy: Survey endorsed by the Italian Association of Radiotherapy and Clinical Oncology (AIRO). Radiother Oncol..

[36] Menon L.K., Richard V., de Mestral C., Baysson H., Wisniak A., Guessous I., Stringhini S., Specchio C.S.G. (2022). Forgoing healthcare during the COVID-19 pandemic in Geneva, Switzerland—A cross-sectional population-based study. Prev. Med..

[37] Xu L., Zhuo L., Zhang J., Yang W., Liu G., Zhan S., Wang S., Xiao H. (2021). Impact of the COVID-19 Pandemic on Outpatient Service in Primary Healthcare Institutions: An Inspiration From Yinchuan of China. Int. J. Health Policy Manag..

[38] Centre for Research on Health and Social Care Management (CERGAS) OASI Report 2021. https://cergas.unibocconi.eu/observatories/oasi_/oasi-report-2021.

[39] Palmer K., Monaco A., Kivipelto M., Onder G., Maggi S., Michel J.P., Prieto R., Sykara G., Donde S. (2020). The potential long-term impact of the COVID-19 outbreak on patients with non-communicable diseases in Europe: Consequences for healthy ageing. Aging Clin. Exp. Res..

[40] Czeisler M.E., Kennedy J.L., Wiley J.F., Facer-Childs E.R., Robbins R., Barger L.K., Czeisler C.A., Rajaratnam S.M.W., Howard M.E. (2021). Delay or avoidance of routine, urgent and emergency medical care due to concerns about COVID-19 in a region with low COVID-19 prevalence: Victoria, Australia. Respirology.

[41] Ganson K.T., Weiser S.D., Tsai A.C., Nagata J.M. (2020). Associations between Anxiety and Depression Symptoms and Medical Care Avoidance during COVID-19. J. Gen. Intern. Med..

[42] Wong S.Y.S., Zhang D., Sit R.W.S., Yip B.H.K., Chung R.Y., Wong C.K.M., Chan D.C.C., Sun W., Kwok K.O., Mercer S.W. (2020). Impact of COVID-19 on loneliness, mental health, and health service utilisation: A prospective cohort study of older adults with multimorbidity in primary care. Br. J. Gen. Pract..

[43] Smolic S., Cipin I., Medimurec P. (2021). Access to healthcare for people aged 50+ in Europe during the COVID-19 outbreak. Eur. J. Ageing.

[44] Amerio A., Aguglia A., Odone A., Gianfredi V., Serafini G., Signorelli C., Amore M. (2020). Covid-19 pandemic impact on mental health of vulnerable populations. Acta Biomed..

[45] Hung K.K., Walline J.H., Chan E.Y.Y., Huang Z., Lo E.S.K., Yeoh E.K., Graham C.A. (2022). Health Service Utilization in Hong Kong During the COVID-19 Pandemic—A Cross-sectional Public Survey. Int. J. Health Policy Manag..

[46] Bertakis K.D., Azari R., Helms L.J., Callahan E.J., Robbins J.A. (2000). Gender differences in the utilization of health care services. J. Fam. Pract..

[47] Hiller J., Schatz K., Drexler H. (2017). Gender influence on health and risk behavior in primary prevention: A systematic review. J. Public Health.

[48] Onder G., Rezza G., Brusaferro S. (2020). Case-Fatality Rate and Characteristics of Patients Dying in Relation to COVID-19 in Italy. JAMA.

[49] Allan I., Ammi M. (2021). Evolution of the determinants of unmet health care needs in a universal health care system: Canada, 2001-2014. Health Econ. Policy Law.

[50] Arnault L., Jusot F., Renaud T. (2021). Economic vulnerability and unmet healthcare needs among the population aged 50 + years during the COVID-19 pandemic in Europe. Eur. J. Ageing.

[51] Gerst-Emerson K., Shovali T.E., Markides K.S. (2014). Loneliness among very old Mexican Americans: Findings from the Hispanic Established Populations Epidemiologic Studies of the Elderly. Arch. Gerontol. Geriatr..

[52] Hajek A., De Bock F., Kretzler B., König H.H. (2021). Factors associated with postponed health checkups during the COVID-19 pandemic in Germany. Public Health.

[53] Gao Y.D., Ding M., Dong X., Zhang J.J., Kursat Azkur A., Azkur D., Gan H., Sun Y.L., Fu W., Li W. (2021). Risk factors for severe and critically ill COVID-19 patients: A review. Allergy.

[54] Topriceanu C.C., Wong A., Moon J.C., Hughes A.D., Bann D., Chaturvedi N., Patalay P., Conti G., Captur G. (2021). Evaluating access to health and care services during lockdown by the COVID-19 survey in five UK national longitudinal studies. BMJ Open.

[55] Michalowsky B., Hoffmann W., Bohlken J., Kostev K. (2021). Effect of the COVID-19 lockdown on disease recognition and utilisation of healthcare services in the older population in Germany: A cross-sectional study. Age Ageing.

[56] ISS La Sorveglianza Passi d’Argento. La Qualità della Vita Vista Dalle Persone con 65 Anni e Più. Epicentro, Istituto Superiore di Sanità. https://www.epicentro.iss.it/passi-argento/.

[57] ISS La Sorveglianza Passi d’Argento. I Dati per l’Italia. Epicentro, Istituto Superiore di Sanità. https://www.epicentro.iss.it/passi-argento/dati/croniche.

[58] McKee M., Kluge H. (2018). Include, invest, innovate: Health systems for prosperity and solidarity. J. Health Serv. Res. Policy.

[59] OECD OECD Regional Well-Being. https://oecdregionalwellbeing.org/ITC4.html.

[60] Atella V., Piano Mortari A., Kopinska J., Belotti F., Lapi F., Cricelli C., Fontana L. (2019). Trends in age-related disease burden and healthcare utilization. Aging Cell.

[61] DiNardo J. (2016). Natural Experiments and Quasi-Natural Experiments. The New Palgrave Dictionary of Economics.

[62] Schmid-Kupke N.K., Matysiak-Klose D., Siedler A., Felgendreff L., Wieler L., Thaiss H.M., Betsch C. (2021). Cancelled routine vaccination appointments due to COVID-19 pandemic in Germany. Vaccine X.

[63] Sabbatucci M., Odone A., Signorelli C., Siddu A., Silenzi A., Maraglino F.P., Rezza G. (2022). Childhood Immunisation Coverage during the COVID-19 Epidemic in Italy. Vaccines.

[64] Wong B.L.H., Maaß L., Vodden A., van Kessel R., Sorbello S., Buttigieg S., Odone A. (2022). The dawn of digital public health in Europe: Implications for public health policy and practice. Lancet Reg. Health Eur..

[65] Chen J., Rizzo J.A., Rodriguez H.P. (2011). The health effects of cost-related treatment delays. Am. J. Med. Qual..

[66] Woolf S.H., Chapman D.A., Sabo R.T., Weinberger D.M., Hill L., Taylor D.D.H. (2020). Excess Deaths From COVID-19 and Other Causes, March–July 2020. JAMA.

[67] González-Touya M., Stoyanova A., Urbanos-Garrido R.M. (2021). COVID-19 and Unmet Healthcare Needs of Older People: Did Inequity Arise in Europe?. Int. J. Environ. Res. Public Health.

