# Supplementary material for: Older Adults’ Access to Care during the COVID-19 Pandemic: Results from the LOckdown and LifeSTyles (LOST) in Lombardia Project
Source: Int J Environ Res Public Health. 2022 Sep 7;19(18):11271. doi: 10.3390/ijerph191811271 (PMC9565221; doi:10.3390/ijerph191811271)
Supplement: Supplementary file 1 [file ijerph-19-11271-s001.zip › Supplementary Table S1.pdf]

Supplementary Table S1. Prevalence of healthcare demand behaviour according to selected characteristics, adjusted prevalence ratios (PRs) and corresponding 95% confidence intervals (CI)*, among the subgroup of 3,521 subjects with at least one chronic condition.

	**Cancellation/postponement of scheduled visits**		**Reduction of ED access**		**Reduction of hospitalisations**	
	Prev. (n)	PR[1] (95% CI)	Prev. (n)	PR[1] (95% CI)	Prev. (n)	PR[1] (95% CI)
**Overall**	26.7 (940)		8.0 (282)		6.1 (217)	
**Sex**						
Women	24.8 (520)	1[2]	7.7 (163)	1[2]	5.8 (124)	1[2]
Men	29.4 (420)	**1.20 (1.07-1.34)**	8.3 (119)	1.12 (0.89-1.41)	6.5 (93)	1.16 (0.89-1.51)
**Age group**						
65-74	24.7 (373)	1[2]	6.4 (97)	1[2]	4.4 (66)	1[2]
≥75	28.3 (567)	**1.13 (1.01-1.27)**	9.2 (185)	**1.56 (1.21-1.99)**	7.5 (151)	**1.90 (1.42-2.55)**
**Marital status**						
Divorced/widowed/single	26.4 (280)	1[2]	7.5 (80)	1[2]	5.5 (60)	1[2]
Married	26.8 (660)	1.15 (0.93-1.42)	8.2 (202)	0.85 (0.59-1.23)	6.4 (157)	0.86 (0.56-1.31)
**Number of household components**						
1	28.2 (230)	1[2]	6.4 (53)	1[2]	4.6 (39)	1[2]
2	27.1 (587)	0.86 (0.68-1.08)	7.7 (168)	**1.58 (1.02-2.45)**	6.2 (134)	**1.76 (1.06-2.94)**
≥3	22.7 (123)	**0.76 (0.58-0.99)**	11.3 (61)	**2.47 (1.55-3.92)**	8.1 (44)	**2.59 (1.50-4.46)**
P for trend		***0.035***		***<0.001***		***<0.001***
**Education level**						
None or primary school	25.7 (180)	1[2]	9.5 (67)	1[2]	6.8 (48)	1
Secondary	25.9 (329)	1.02 (0.87-1.20)	6.6 (84)	0.74 (0.54-1.01)	5.5 (70)	0.86 (0.60-1.24)
High school/Degree	27.8 (431)	1.07 (0.91-1.25)	8.4 (131)	0.94 (0.70-1.27)	6.3 (99)	1.02 (0.72-1.45)
P for trend		*0.065*		*0.917*		*0.711*
**Self-reported economic status**						
Low	21.7 (140)	1[2]	10.5 (68)	1[2]	6.5 (42)	1[2]
Medium	26.6 (691)	**1.31 (1.11-1.55)**	7.2 (189)	0.85 (0.57-1.27)	5.8 (152)	0.73 (0.48-1.11)
High	39.5 (109)	**1.89 (1.53-2.34)**	9.1 (25)	1.26 (0.80-1.98)	8.4 (23)	0.80 (0.48-1.33)
P for trend		***<0.001***		*0.074*		*0.582*
**Number of comorbidities**						
1	20.9 (267)	1[2]	7.0 (90)	1[2]	5.4 (69)	1[2]
2	28.9 (414)	**1.44 (1.26-1.65)**	7.6 (109)	1.11 (0.85-1.46)	5.5 (79)	1.02 (0.74-1.39)
≥3	32.3 (259)	**1.57 (1.35-1.82)**	10.2 (83)	**1.47 (1.10-1.96)**	8.5 (69)	**1.55 (1.11-2.15)**
P for trend		***<0.001***		***0.012***		***0.013***
**COVID-19 infection**						
No	26.6 (897)	1[2]	7.9 (266)	1[2]	6.0 (205)	1[2]
Yes	28.5 (43)	0.92 (0.72-1.19)	10.5 (16)	1.31 (0.81-2.11)	7.9 (12)	1.3 (0.75-2.27)
**Anxiety symptoms (GAD-2) in 2019**						
No (<3)	26.9 (826)	1[2]	8.1 (249)	1[2]	6.2 (190)	1[2]
Yes (≥3)	25.0 (114)	0.95 (0.80-1.14)	7.2 (33)	0.91 (0.63-1.3)	5.8 (27)	0.96 (0.65-1.44)
**Change in anxiety symptoms 2020 vs 2019**						
Unchanged (<3 in both periods)	26.2 (636)	1[2]	7.6 (185)	1[2]	5.9 (144)	1[2]
Unchanged (≥3 in both periods)	25.3 (102)	1.00 (0.83-1.20)	6.6 (27)	0.9 (0.6-1.34)	5.6 (23)	0.97 (0.63-1.51)
Worsened	30.0 (190)	**1.19 (1.04-1.36)**	10.1 (64)	**1.33 (1.01-1.75)**	7.2 (46)	1.22 (0.88-1.69)
Improved	22.9 (12)	0.99 (0.60-1.63)	11.1 (6)	1.52 (0.70-3.29)	7.4 (4)	1.32 (0.50-3.48)
**Depressive symptoms (PHQ-2) in 2019**						
No (<3)	26.9 (861)	1[2]	8.2 (263)	1[2]	6.3 (203)	1[2]
Yes (≥3)	24.9 (79)	0.95 (0.77-1.17)	5.8 (19)	0.72 (0.45-1.15)	4.3 (14)	0.68 (0.39-1.17)
**Change in depressive symptoms 2020 vs 2019**						
Unchanged (<3 in both periods)	26.2 (740)	1[2]	8.1 (228)	1[2]	6.2 (177)	1[2]
Unchanged (≥3 in both periods)	24.9 (66)	0.96 (0.77-1.20)	5.1 (14)	0.64 (0.37-1.10)	3.6 (10)	0.57 (0.30-1.09)
Worsened	31.7 (121)	**1.20 (1.02-1.40)**	9.1 (35)	1.14 (0.81-1.60)	6.7 (26)	1.10 (0.73-1.65)
Improved	24.6 (13)	1.06 (0.65-1.72)	9.2 (5)	1.25 (0.53-2.95)	7.7 (4)	1.32 (0.51-3.39)

GAD-2: Generalized Anxiety Disorder; PHQ-2: Patient Health Questionnaire.

[1]Estimated through weighted log-binomial regression models adjusted by sex, age group, marital status, education level, number of household components, self-reported economic status, number of chronic diseases, and self-reported COVID-19 infection.

[2]Reference category.
